# Natural clusters of tuberous sclerosis complex (TSC)-associated neuropsychiatric disorders (TAND): new findings from the TOSCA TAND research project

**DOI:** 10.1186/s11689-020-09327-0

**Published:** 2020-09-01

**Authors:** Petrus J. de Vries, Elena Belousova, Mirjana P. Benedik, Tom Carter, Vincent Cottin, Paolo Curatolo, Lisa D’Amato, Guillaume Beure d’Augères, José C. Ferreira, Martha Feucht, Carla Fladrowski, Christoph Hertzberg, Sergiusz Jozwiak, John A. Lawson, Alfons Macaya, Ruben Marques, Rima Nabbout, Finbar O’Callaghan, Jiong Qin, Valentin Sander, Matthias Sauter, Seema Shah, Yukitoshi Takahashi, Renaud Touraine, Sotiris Youroukos, Bernard Zonnenberg, J. Chris Kingswood, Anna C. Jansen, on behalf of TOSCA Consortium and TOSCA Investigators, Nobuo Shinohara, Shigeo Horie, Masaya Kubota, Jun Tohyama, Katsumi Imai, Mari Kaneda, Hideo Kaneko, Yasushi Uchida, Tomoko Kirino, Shoichi Endo, Yoshikazu Inoue, Katsuhisa Uruno, Ayse Serdaroglu, Zuhal Yapici, Banu Anlar, Sakir Altunbasak, Olga Lvova, Oleg Valeryevich Belyaev, Oleg Agranovich, Elena Vladislavovna Levitina, Yulia Vladimirovna Maksimova, Antonina Karas, Yuwu Jiang, Liping Zou, Kaifeng Xu, Yushi Zhang, Guoming Luan, Yuqin Zhang, Yi Wang, Meiling Jin, Dingwei Ye, Weiping Liao, Liemin Zhou, Jie Liu, Jianxiang Liao, Bo Yan, Yanchun Deng, Li Jiang, Zhisheng Liu, Shaoping Huang, Hua Li, Kijoong Kim, Pei-Lung Chen, Hsiu-Fen Lee, Jeng-Dau Tsai, Ching-Shiang Chi, Chao-Ching Huang, Kate Riney, Deborah Yates, Patrick Kwan, Surachai Likasitwattanakul, Charcrin Nabangchang, Lunliya Thampratankul Krisnachai Chomtho, Kamornwan Katanyuwong, Somjit Sriudomkajorn, Jo Wilmshurst, Reeval Segel, Tal Gilboa, Michal Tzadok, Aviva Fattal-Valevski, Panagiotis Papathanasopoulos, Antigone Syrigou Papavasiliou, Stylianos Giannakodimos, Stylianos Gatzonis, Evangelos Pavlou, Meropi Tzoufi, A. M. H. Vergeer, Marc Dhooghe, Hélène Verhelst, Filip Roelens, Marie Cecile Nassogne, Pierre Defresne, Liesbeth De Waele, Patricia Leroy, Nathalie Demonceau, Benjamin Legros, Patrick Van Bogaert, Berten Ceulemans, Lina Dom, Pierre Castelnau, Anne De Saint Martin, Audrey Riquet, Mathieu Milh, Claude Cances, Jean-Michel Pedespan, Dorothee Ville, Agathe Roubertie, Stéphane Auvin, Patrick Berquin, Christian Richelme, Catherine Allaire, Sophie Gueden, Sylvie Nguyen The Tich, Bertrand Godet, Maria Luz Ruiz Falco Rojas, Jaume Campistol Planas, Antonio Martinez Bermejo, Patricia Smeyers Dura, Susana Roldan Aparicio, Maria Jesus Martinez Gonzalez, Javier Lopez Pison, Manuel Oscar Blanco Barca, Eduardo Lopez Laso, Olga Alonso Luengo, Francisco Javier Aguirre Rodriguez, Ignacio Malaga Dieguez, Ana Camacho Salas, Itxaso Marti Carrera, Eduardo Martinez Salcedo, Maria Eugenia Yoldi Petri, Ramon Cancho Candela, Ines da Conceicao Carrilho, Jose Pedro Vieira, José Paulo da Silva Oliveira Monteiro, Miguel Jorge Santos de Oliveira Ferreira Leao, Catarina Sofia Marceano Ribeiro Luis, Carla Pires Mendonca, Milda Endziniene, Jurgis Strautmanis, Inga Talvik, Maria Paola Canevini, Antonio Gambardella, Dario Pruna, Salvatore Buono, Elena Fontana, Bernardo Dalla Bernardina, Carmen Burloiu, Iuliu Stefan Bacos Cosma, Mihaela Adela Vintan, Laura Popescu, Karel Zitterbart, Jaroslava Payerova, Ladislav Bratsky, Zuzana Zilinska, Ursula Gruber-Sedlmayr, Matthias Baumann, Edda Haberlandt, Kevin Rostasy, Ekaterina Pataraia, Frances Elmslie, Clare Ann Johnston, Pamela Crawford, Peter Uldall, Maria Dahlin, Paul Uvebrant, Olof Rask, Marit Bjoernvold, Eylert Brodtkorb, Andreas Sloerdahl, Ragnar Solhoff, Martine Sofie Gilje Jaatun, Marek Mandera, Elzbieta Janina Radzikowska, Mariusz Wysocki, Michael Fischereder, Gerhard Kurlemann, Bernd Wilken, Adelheid Wiemer-Kruel, Klemens Budde, Klaus Marquard, Markus Knuf, Andreas Hahn, Hans Hartmann, Andreas Merkenschlager, Regina Trollmann

**Affiliations:** 1 Division of Child and Adolescent Psychiatry, University of Cape Town, 46 Sawkins Road, Rondebosch, Cape Town, 7700 South Africa; 2 Research and Clinical Institute of Pediatrics, Pirogov Russian National Research Medical University, Moscow, Russian Federation; 3 SPS Pediatrična Klinika, Ljubljana, Slovenia; 4 TSA Tuberous Sclerosis Association, Nottingham, UK; 5 Hôpital Louis Pradel, Claude Bernard University Lyon 1, Lyon, France; 6 Tor Vergata University Hospital, Rome, Italy; 7 Novartis Farma S.p.A., Origgio, Italy; 8 Association Sclérose Tubéreuse de Bourneville, Gradignan, France; 9 Centro Hospitalar Lisboa Ocidental, Lisbon, Portugal; 10 Universitätsklinik für Kinder-und Jugendheilkunde, Affiliated Partner of the ERN EpiCARE, Vienna, Austria; 11 Associazione Sclerosi Tuberosa ONLUS, Milan, Italy; 12 European Tuberous Sclerosis Complex Association, In den Birken, Datteln, Germany; 13 Vivantes-Klinikum Neukölln, Berlin, Germany; 14 Department of Child Neurology, Warsaw Medical University, Warsaw, Poland; 15 Department of Neurology and Epileptology, The Children’s Memorial Health Institute, Warsaw, Poland; 16 The Tuberous Sclerosis Multidisciplinary Management Clinic, Sydney Children’s Hospital, Randwick, NSW Australia; 17 Hospital Universitari Vall d’Hebron, Barcelona, Spain; 18 Institute of Biomedicine (IBIOMED), University of Leon, León, Spain; 19 Department of Pediatric Neurology, Necker Enfants Malades Hospital, Paris Descartes University, Paris, France; 20 Institute of Child Health, University College London, London, UK; 21 Department of Pediatrics, Peking University People’s Hospital (PKUPH), Beijing, China; 22 Tallinn Children Hospital, Tallinn, Estonia; 23 Klinikverbund Kempten-Oberallgäu gGmbH, Kempten, Germany; 24 Novartis Healthcare Pvt. Ltd., Hyderabad, India; 25 National Epilepsy Center, Shizuoka Institute of Epilepsy and Neurological Disorders, NHO, 886 Urushiyama Aoi-ku, Shizuoka, Japan; 26 Department of Genetics, CHU-Hôpital Nord, Saint Etienne, France; 27 St. Sophia Children’s Hospital, Athens, Greece; 28 University Medical Center, Utrecht, Netherlands; 29 Cardiology Clinical Academic Group, Molecular and Clinical Sciences Research Centre, St Georges University of London, London, SW17 0RE UK; 30 Pediatric Neurology Unit, Department of Pediatrics, UZ Brussel VUB, Brussels, Belgium

**Keywords:** ASD, Cluster analysis, Factor analysis, Natural TAND clusters, TAND, Tuberous sclerosis complex, TOSCA, Registry, Neuropsychiatric

## Abstract

**Background:**

Tuberous sclerosis complex (TSC)-associated neuropsychiatric disorders (TAND) have unique, individual patterns that pose significant challenges for diagnosis, psycho-education, and intervention planning. A recent study suggested that it may be feasible to use TAND Checklist data and data-driven methods to generate natural TAND clusters. However, the study had a small sample size and data from only two countries. Here, we investigated the replicability of identifying natural TAND clusters from a larger and more diverse sample from the TOSCA study.

**Methods:**

As part of the TOSCA international TSC registry study, this embedded research project collected TAND Checklist data from individuals with TSC. Correlation coefficients were calculated for TAND variables to generate a correlation matrix. Hierarchical cluster and factor analysis methods were used for data reduction and identification of natural TAND clusters.

**Results:**

A total of 85 individuals with TSC (female:male, 40:45) from 7 countries were enrolled. Cluster analysis grouped the TAND variables into 6 clusters: a scholastic cluster (reading, writing, spelling, mathematics, visuo-spatial difficulties, disorientation), a hyperactive/impulsive cluster (hyperactivity, impulsivity, self-injurious behavior), a mood/anxiety cluster (anxiety, depressed mood, sleep difficulties, shyness), a neuropsychological cluster (attention/concentration difficulties, memory, attention, dual/multi-tasking, executive skills deficits), a dysregulated behavior cluster (mood swings, aggressive outbursts, temper tantrums), and an autism spectrum disorder (ASD)-like cluster (delayed language, poor eye contact, repetitive behaviors, unusual use of language, inflexibility, difficulties associated with eating). The natural clusters mapped reasonably well onto the six-factor solution generated. Comparison between cluster and factor solutions from this study and the earlier feasibility study showed significant similarity, particularly in cluster solutions.

**Conclusions:**

Results from this TOSCA research project in an independent international data set showed that the combination of cluster analysis and factor analysis may be able to identify clinically meaningful natural TAND clusters. Findings were remarkably similar to those identified in the earlier feasibility study, supporting the potential robustness of these natural TAND clusters. Further steps should include examination of larger samples, investigation of internal consistency, and evaluation of the robustness of the proposed natural clusters.

## Background

Tuberous sclerosis complex (TSC) is a complex multisystem genetic disorder with a vast and variable age-related presentation of physical and neuropsychiatric manifestations [1–3]. It is associated with a substantial economic and psychosocial burden on the affected individuals and their families [1, 4–7].

In spite of the high rates and burden of neuropsychiatric manifestations in individuals with TSC, a 2010 study from the UK reported that only 18% of all families had ever received any of the recommended evaluations or treatments for the range of neuropsychiatric manifestations [8]. These findings suggested a large assessment and treatment gap in TSC. In order to reduce this gap, the Neuropsychiatry Panel of the International Consensus Guidelines Group coined the term TAND (TSC-associated neuropsychiatric disorders) in 2012 [9] and presented a standardized nomenclature to describe the range of neuropsychiatric manifestations observed in TSC across six levels—behavioral, psychiatric, intellectual, academic, neuropsychological, and psychosocial. The Neuropsychiatry Panel also recommended that all individuals with TSC should be screened for TAND on an annual basis [9]. In order to support screening for TAND, a TAND Checklist was developed through a participatory research strategy and pilot validated [10, 11].

Individuals with TSC have unique and highly variable TAND profiles. This uniqueness and multi-dimensionality of TAND often lead to ‘treatment paralysis’ where most clinical teams feel overwhelmed by the complexity of the neuropsychiatric presentations of their patients with TSC, thus posing a significant challenge to clinicians for diagnosis, psycho-education, and intervention planning [12, 13]. To reduce the assessment gap and treatment paralysis seen in the TSC community, the possibility of identifying “natural clusters” of the TAND phenomena was hypothesized by Leclezio and de Vries [12]. They proposed that, if data-driven strategies could identify a manageable number of clusters, this could reduce the assessment and treatment gap by providing clinical next steps [13]. The researchers proposed this to be an essential first step towards personalisation of clinical concerns, guiding the generation of evidence-based treatments for TAND and adding precision to training and fundamental neuroscience research [13].

In a feasibility study, Leclezio and colleagues explored methods that may identify natural clusters [14]. Findings identified WARD’s cluster analysis and exploratory factor analysis as potential methods and produced six natural clusters with good face validity. However, the study had a small sample size (*n* = 56) and included patients from only two countries (South Africa and Australia). Given the highly heterogeneous nature of TAND manifestations, it was therefore not clear to what extent the six identified clusters would be replicable.

In this study, we set out to examine a new sample of individuals with TSC across ages and abilities from seven countries to determine whether data reduction methods would be able to replicate and extend the findings from the feasibility study performed by Leclezio et al. [14].

## Methods

### Design

The detailed methodology of the overall TOSCA clinical study has been published previously [15]. In brief, TOSCA was a non-interventional, multicenter, natural history registry of individuals with TSC. The study was designed with a “core” section and six research projects, each focusing on a specific area of TSC—subependymal giant cell astrocytoma, renal angiomyolipoma, genetics, epilepsy, quality of life, and TAND. Here, we present data on the research project focusing on TAND.

### Subjects and procedures for this research project

All centers participating in the TOSCA clinical study were invited to participate in the TAND research project. Centers from seven countries opted to participate. All TOSCA participants from these countries were therefore invited to participate in this study. Upon provision of a dedicated informed consent for the TAND research project, the TAND Checklist was administered to individuals with TSC or their caregivers by a study physician [10]. The TAND Checklist follows the neuropsychiatric levels of investigation outlined previously [10, 11] and consists of the following 12 sections: (1) basic developmental milestones; (2) current level of functioning; (3) behavioral difficulties; (4) psychiatric disorders diagnosed; (5) intellectual ability; (6) academic difficulties; (7) neuropsychological deficits; (8) psychosocial functioning; (9) parent, caregiver, or self-rating of the impact of TAND; (10) prioritization list; (11) additional concerns; and (12) health care professional rating of the impact of TAND. The questions require simple yes or no responses in most sections.

### Data analysis

In contrast to “hypothesis-testing” statistical approaches where data are analyzed in relation to an a priori prediction, unsupervised learning or data-driven methods searches for previously undetected patterns or groupings in a dataset without any a priori rules, predictions, or labels to data. In this study, we used cluster analysis and factor analysis, two unsupervised learning/data-driven statistical methods, to help understand the complex TAND data. The objectives of cluster and factor analysis methods are, however, different. Cluster analysis aims to group observations (e.g., a sample of subjects or variables) into distinct groups in a way that objects in that group are more similar to each other than to those in other clusters or groups. Many different methods are used for cluster analysis. In the proof-of-principle study by Leclezio et al. [14], a wide range of cluster analysis methods were explored and the WARD method was identified as the most suitable method for the TAND Checklist data used. WARD is a hierarchical cluster analysis method. The method starts with each object as a separate cluster. At each sequential step, the two closest clusters are merged. The WARD method bases the closeness of clusters on within cluster variance. The sequential merging is typically visualized in a dendrogram (or hierarchical tree).

In contrast to the intuitive stepwise WARD clustering algorithm, factor analysis is based on fitting a model to the data. Factor analysis is typically used as a data reduction method to reduce a larger set of variables into a much smaller number of factors. The model assumes a few unobservable “latent (or underlying) factors” in the data. Factor analysis uses the correlations between variables (e.g., TAND checklist items) to identify latent factors representing a group of highly correlated variables. (A group of highly correlated variables will tend to vary jointly, thus reducing the within group variance). Factor analysis data are typically visualized as correlation matrices showing the factor loadings of items included in each factor. Factor score plots represent a different visualization method and show how factor scores contribute to each factor. In the Leclezio et al. study [14], a range of exploratory factor analysis methods were used for extraction and rotation of data to find a factor solution that best matched the cluster analysis method. Ultimately both methods (cluster and factor analysis) group similar items, but follow very different approaches. In general, where the two methods converge on the same findings, this allows one to place increased confidence in those findings.

In order to replicate the proof-of-concept work by Leclezio et al. [14], we included exactly the same variables for analysis. The following sections of the TAND Checklist were included: Section 3, behavioral challenges (19 questions/variables); Section 6, academic skills (four variables); and Section 7, neuropsychological skills (six variables). In the original study, variables were included that were (a) descriptive of observed phenomena, e.g., the behavioral, scholastic or neuropsychological levels, and (b) that could have been answered without access to specialist care (e.g., no need for diagnosis or formal testing). Given that all the variables had binary (yes/no), a scoring coefficient was used to compute a correlation matrix for the variables of interest. In case of missing values, variables were omitted pairwise in correlation computations. Hierarchical cluster analysis was used to identify natural clusters and to generate a clustering tree (dendrogram) visually representing the merging of TAND variables and suggesting a suitable number of clusters. Factor analysis was performed for data reduction based on correlation between the variables. The number of factors in the model was matched to the number of natural clusters identified. Cluster and factor solutions were compared to examine overlap between the two data reduction methods. In the absence of access to data to perform a direct statistical comparison, a narrative comparison was made of the cluster and factor solutions between this study and the feasibility study [14].

## Results

Eighty-five individuals (31 adults and 54 children) from 7 countries were enrolled in this research project. The demographic characteristics of the participants are shown in Table 1. Median age at consent was 14 years (mean, 17.8 years; range, 2–72 years).

**Table 1:** Demographic characteristics

	Overall participants (*N* = 85)
**Sex,** ***n*** **(%)**	
Male	40 (47.1)
Female	45 (52.9)
**Age strata (years),** ***n*** **(%)**	
≤ 2	1 (1.2)
> 2 to ≤ 5	11 (12.9)
> 5 to ≤ 9	16 (18.8)
> 9 to ≤14	17 (20.0)
> 14 to < 18	9 (10.6)
≥ 18 to ≤ 40	22 (25.9)
> 40	9 (10.6)
**Age at consent, years**	
Mean (SD)	17.8 (14.57)
Median (range)	14 (2–72)
**Country of residence,** ***n*** **(%)**	
Belgium	18 (21.2)
France	33 (38.8)
Germany	7 (8.2)
Spain	7 (8.2)
UK	4 (4.7)
Japan	15 (17.6)
Turkey	1 (1.2)

*SD* standard deviation, *UK* United Kingdom

### Cluster analysis and exploratory factor analysis

**Fig. 1:**
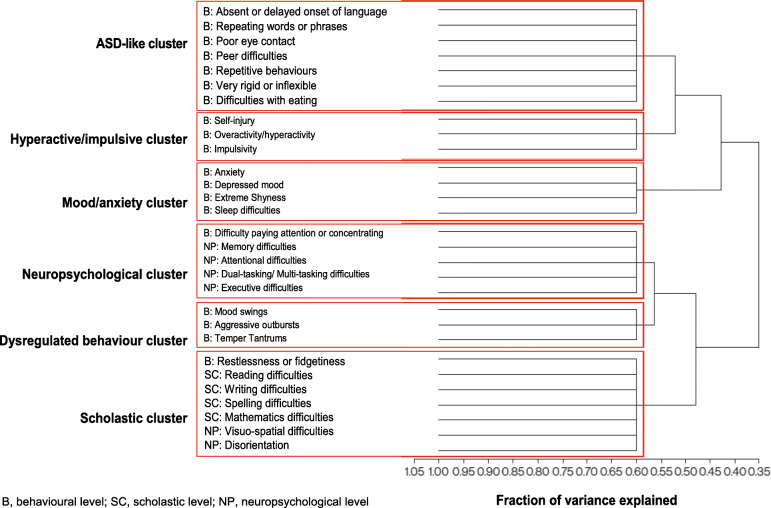
Dendrogram of natural TAND clusters. Hierarchical cluster analysis using the WARD method produced six natural TAND clusters

**Fig. 2:**
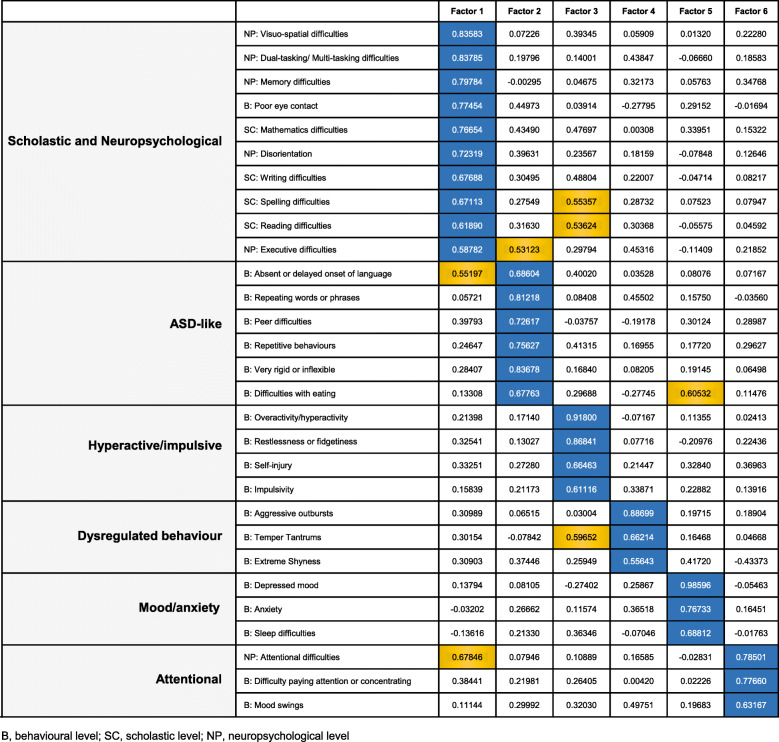
Exploratory factor analysis results of a six-factor solution to identify the latent constructs underlying the TAND variables. The figure shows the rotated factor pattern using the Varimax method. Coefficients in blue represent the largest coefficient values for each variable across all 6 factors. All other coefficients with values > 0.5 are shown in yellow

**Fig. 3:**
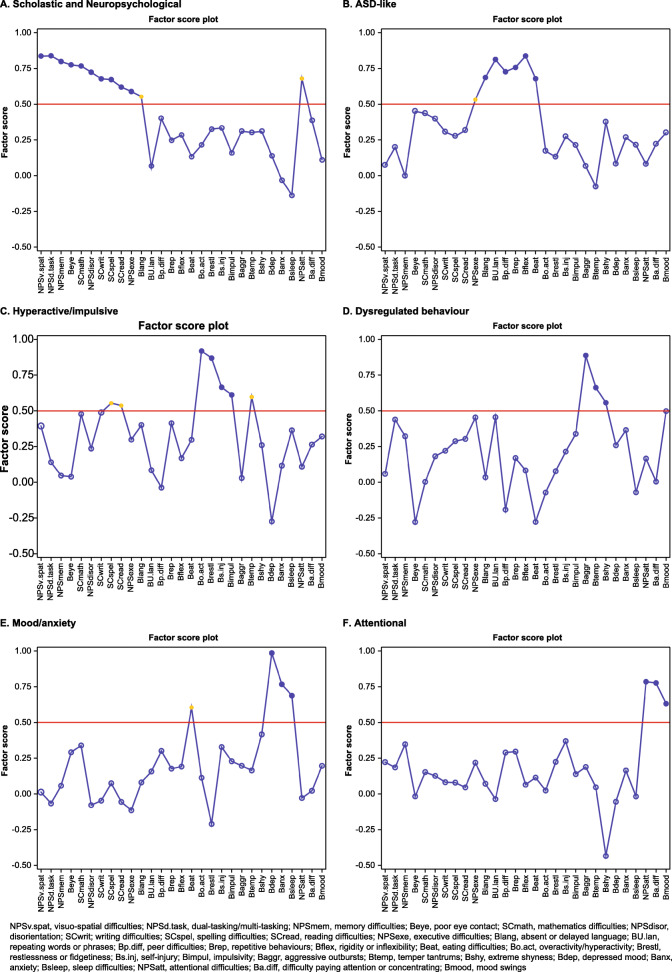
Visualization of the factor score graph showing factor scores of individual TAND variables in relation to the six-factor solution derived from exploratory factor analysis. The closer a factor score is to + 1 the stronger the influence of the factor is on that variable. Solid blue dots represent the largest coefficient values for each variable across all 6 factors and solid yellow dots represent all other coefficients with values > 0.5. Blue circles represent coefficients with values < 0.5

Hierarchical clustering identified six natural clusters of TAND variables as the most parsimonious solution. A dendrogram detailing these six natural clusters is shown in Fig. 1. The first cluster included difficulties with reading, writing, spelling, mathematics, visuo-spatial tasks, restlessness, and disorientation, suggesting a natural “scholastic” cluster. The second cluster included mood swings, aggressive outbursts, and temper tantrums, suggesting a natural “dysregulated behavior” cluster. The third cluster included difficulties in attention/concentration, deficits in memory, neuropsychological attention deficits, dual/multi-tasking, and executive skills. These characteristics suggested a natural “neuropsychological” cluster. The fourth cluster included anxiety, depressed mood, sleep difficulties, and extreme shyness, suggesting a natural “mood/anxiety” cluster. The fifth cluster included self-injurious behavior, hyperactivity, and impulsivity, suggesting a natural “hyperactive/impulsive” cluster. The sixth cluster included delayed language, poor eye contact, repetitive behaviors, unusual use of language, rigidity or inflexibility, and difficulties associated with eating. These characteristics suggested a natural “autism spectrum disorder (ASD)-like” cluster. The exploratory factor analysis findings are shown in Figs. 2 and 3.

### Comparison of cluster analysis and factor analysis

**Fig. 4:**
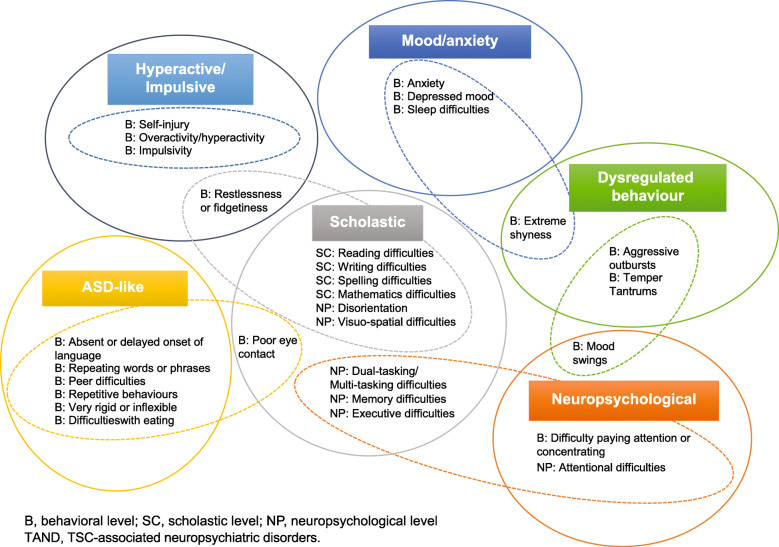
Comparison of cluster analysis and exploratory factor analysis to show the overlap between cluster and factor solutions. Dotted lines indicate natural TAND clusters; solid lines show factor analysis solutions

The similarities and differences between cluster analysis and exploratory factor analysis are shown in Fig. 4. The six factors mapped reasonably well onto the natural clusters identified as linked to scholastic skills, ASD, dysregulated behavior, neuropsychological deficits, hyperactive/impulsive behaviors, and mood/anxiety. With the exception of poor eye contact, there was a 100% overlap between the “ASD-like” natural TAND cluster and the ASD-related factor solution (delayed language, repetitive behaviors, unusual use of language, rigidity or inflexibility, and difficulties associated with eating). In the hyperactive/impulsive natural TAND cluster, factor analysis included one additional characteristic (restlessness), but the other items were identical. In the dysregulated behavior natural TAND cluster, factor analysis included one additional characteristic (extreme shyness), and grouped mood swings with neuropsychological attention deficits and behavioral attention deficits. Aggressive outbursts and temper tantrums were both present in the dysregulated behavior cluster and factor. With regard to the mood/anxiety natural TAND cluster, factor analysis had grouped extreme shyness with other items in the dysregulated behavior cluster. Other mood/anxiety items were the same in the cluster and factor solutions. In the scholastic natural TAND cluster, factor analysis included three neuropsychological variables (dual/multi-tasking, memory, and executive skills), but the other items were identical. A separate “neuropsychological attentional factor” with high cross-loading onto the other neuropsychological variables and the neuropsychological cluster was identified.

### Narrative comparison of findings between the feasibility study (Leclezio et al. 2018) and the present study

#### Cluster solutions

The majority of items from the TAND Checklist were grouped similarly between the two studies. Both the feasibility study and this study showed six natural clusters, with identical findings for the dysregulated behavior and mood/anxiety clusters between the studies (Table 2). In the ASD-like cluster, five variables (language, unusual language, repetitive behavior, poor eye contact, and eating difficulties) were identical between the studies. However, this study also included peer difficulties and inflexibility with the ASD-like cluster. This grouping has good face validity in relation to the clinical characteristics of ASD. In terms of the scholastic cluster, all core scholastic items (difficulties with reading, writing, spelling, mathematical problems) were grouped together in the feasibility study and in this study. However, two items that appeared more neuropsychological in construct (disorientation and visuo-spatial deficits) were also grouped in the scholastic cluster in the present study. In the hyperactive/impulsive cluster, overactivity, and impulsivity were grouped together in the feasibility study and in this study, but restlessness (grouped with hyperactive/impulsive behaviors in the feasibility study) was clustered in the scholastic cluster in this study. In both studies, attention deficits (behavioral level and neuropsychological attention deficits) clustered separately from the overactive/impulsive items.

**Table 2:** Comparison of clusters and factors between the feasibility study (Leclezio et al. 2018) and this study (the replication study)

Clusters and factors	Variables		
	Both feasibility and replication studies	Replication study (current study)	Feasibility study (Leclezio et al. 2018)
**TAND clusters**			
1. Mood/anxiety	Anxiety (Banx) Depressed mood (Bdep) Extreme shyness (Bshy) Sleep difficulties (Bsleep)	-	-
2. Dysregulated behavior	Mood swings (Bmood) Aggressive outbursts (Baggr) Temper tantrums (Btemp)	-	-
3. ASD-like	Absent or delayed language (Blang) Repeating words or phrases (BU.lan) Poor eye contact (Beye) Repetitive behaviors (Brep) Difficulties with eating (Beat)	Peer difficulties (Bp.diff) Rigidity/inflexibility (Bflex)	Self-injury (Bs.inj) Visuo-spatial difficulties (NPSv.spat)
4. Hyperactive/impulsive	Overactivity (Bo.act) Impulsivity (Bimpul)	Self-injury (Bs.inj)	Rigidity/inflexibility (Bflex) Restlessness (Brestl)
5. Neuropsychological	Difficulty paying attention (Ba.diff) Memory difficulties (NPSmem) Attention difficulties (NPSatt) Dual-tasking difficulties (NPSd.task) Executive difficulties (NPSexe)	-	Visuo-spatial difficulties (NPSdisor) Peer difficulties (Bp.diff)
6. Scholastic	Reading difficulties (SCread) Writing difficulties (SCwrit) Spelling difficulties (SCspel) Mathematics difficulties (SCmath)	Restlessness (Brestl) Visuo-spatial difficulties (NPSv.spat) Disorientation (NPSdisor)	-
**TAND factors**			
1. Scholastic and Neuropsychological	Dueal-task difficulties (NPSd.task) Executive difficulties (NPSexe) Mathematics difficulties (SCmath) Reading difficulties (SCread) Writing difficulties (SCwrit) Spelling difficulties (SCspel)	Visuo-spatial difficulties (NPSv.spat) Memory difficulties (NPSmem) Disorientation (NPSdisor) Poor eye contact (Beye)	Attention difficulties (Ba.diff) Neuropsychological attention difficulties (NPSatt)
2. ASD-like	Absent or delayed language (Blang) Repeating words or phrases (BU.lan) Peer difficulties (Bp.diff) Repetitive behaviors (Brep) Eating difficulties (Beat)	Rigidity/inflexibility (Bflex)	Visuo-spatial difficulties (NPSv.spat) Disorientation (NPSdisor) Self-injury (Bs.inj) Poor eye contact (Beye)
3. Hyperactive/impulsive	Restlessness (Brestl) Overactivity (Bo.act) Impulsivity (Bimpul)	Self-injury (Bs.inj)	Rigidity/inflexibility (Bflex)
4. Dysregulated behavior	Aggressive outbursts (Baggr) Temper tantrums (Btemp)	Extreme shyness (Bshy)	Anxiety (Banx) Mood swings (Bmood)
5. Mood/anxiety	Depressed mood (Bdep) Sleep difficulties (Bsleep)	Anxiety (Banx)	Memory difficulties (NPSmem) Extreme shyness (Bshy)
6. Attentional	-NA-	Neuropsychological attention difficulties (NPSatt) Attention difficulties (Ba.diff) Mood swings (Bmood)	-NA-

*ASD* autism spectrum disorder

The columns show all TAND Checklist items included in the study and the abbreviation for each variable in parenthesis e.g. Anxiety (Banx)

#### Factor solutions

We observed less consistency in factor solutions between the two studies. In the ASD-like factor of this study, almost all the variables were identical to those in the feasibility study, except that our factor analysis excluded self-injury, disorientation, poor eye contact, and difficulty in visuo-spatial tasks, and included inflexibility in the factor (Table 2). In the overactive/impulsive factor, three variables (overactive, impulsive, and restlessness) were identical, but inflexibility and self-injury grouped with different factors. Both dysregulated behavior and mood/anxiety factors had almost identical variables, apart from anxiety and extreme shyness that switched factors between the studies. The mood/anxiety factor in the present study excluded memory. In this study, we observed a combined “scholastic and neuropsychological” factor and a new “attentional” factor that included behavioral attention deficits, neuropsychological attention deficits, and mood swings.

## Discussion

Identification of natural TAND clusters through data-driven methods has been proposed as a potential solution for the “treatment paralysis” seen in TSC, given the highly variable and apparently unique nature of TAND profiles in individuals. In a proof-of-principle study, Leclezio, Gardner, and de Vries showed the feasibility of using data reduction methods in TAND and identified six putative natural clusters [14]. However, the sample size of the Leclezio study was very small, and individuals were recruited from only two countries. Given these limitations and the highly heterogeneous nature of TSC, we set out to replicate the feasibility findings in a larger sample of 85 individuals, including children, from seven countries. We observed six natural TAND clusters (scholastic, ASD-like, dysregulated behavior, neuropsychological, overactive/impulsive, and mood/anxiety). These were remarkably similar to those identified by Leclezio et al. in the feasibility study [14], but had more mixed results in factor solutions, thus providing partial replication of the finding of potential natural TAND clusters. However, while some items were clearly differently grouped using data-driven strategies between the feasibility study and this study, many similarities were seen, suggesting that, in spite of the vast heterogeneity of TAND, there may be robust natural clusters of TAND manifestations that should be explored further in larger-scale studies [16–18].

Currently, many families and clinical teams are unaware of which of all the possible TAND manifestations to look out for and how to provide appropriate evidence-based, next-step interventions. If a limited number of natural clusters are confirmed, clinical monitoring, and next steps of psycho-education and intervention for six or so clusters of difficulties would be much more feasible. For instance, it may be possible then to develop modular training based on specific clusters, such as specific programs for dysregulated behavior in TSC or for mood/anxiety cluster features.

It was of interest that some of the natural clustering was in groups that make intuitive diagnostic sense from clinical criteria, such as the ASD-like cluster. TSC is known to be one of the medical conditions most strongly associated with ASD [6]. However, it was also interesting to observe that the hyperactive/impulsive features did not cluster with the inattention features, in contrast with the typical clinical grouping of manifestations associated with attention deficit/hyperactivity disorder (ADHD). In both the feasibility and this study, behavioral attention deficits were more likely to cluster with neuropsychological attention-executive skill deficits. All these proposals will require further evaluation in larger-scale studies.

For the purposes of this early-phase replication study, we wanted to see if, first, we were able to identify robust methodologies and whether they would replicate in an independent sample, and second, whether natural clusters could be identified even in the absence of age and intellectual ability data. The association between age and intellectual ability on TAND clusters, however, raises interesting conceptual and empirical questions. It is likely that TAND cluster profiles may emerge or change over time. For instance, the scholastic cluster is likely not to be relevant in the first few years of life. Similarly, intellectual ability may be a very strong marker of the likelihood of TAND clusters. These important questions will require larger-scale and longitudinal datasets.

In comparison to the feasibility study [14] where only English-speaking participants were used, we deliberately aimed to include a more culturally and linguistically diverse sample to examine the robustness of the putative TAND clusters identified. The sample therefore included French, Dutch, English, German, Spanish, Turkish, and Japanese participants. The TAND Checklist has been translated and authorized in 17 languages to date, and where available, those language versions were used. Larger-scale studies may allow for a comparison of TAND cluster profiles in different cultural and language groups. However, to date, there are no clinical suggestions that TAND manifestations have differential cultural expression.

### Limitations and next steps

There are several potential limitations to this study. We acknowledge that, even though this study sample was larger and more diverse than that of the feasibility study, the sample size was still small, even for a rare disease. We were aiming to recruit from a large natural history study (TOSCA study) and were therefore hopeful to include a much larger sample for this study. However, given that it was embedded in an industry-funded observational trial, a formal procedure for opting in at a country level was required. Where countries opted in, all participants at centers were included. While we therefore acknowledge an “administrative” bias in recruitment, we have no reason to suspect a clinical ascertainment bias, given that all subjects from participating centers had a TAND Checklist completed.

Interestingly, there is no consensus in the literature about the required sample size for cluster analysis, and a number of small-scale studies such as ours have identified meaningful natural clusters [19]. Some authors have suggested a minimum sample size of *n* = 100, while others emphasized the importance of an optimal variable/subject ratio with a 1:10 ratio (1 variable to 10 subjects) as most stringent suggestion [20]. Given the differences observed between the feasibility and replication data sets, we propose that it would be important to proceed to examination of larger-scale samples, ideally in excess of the 1/10 (variable/subject) ratio. Secondly, apart from cluster and factor analysis, it would be important to evaluate the internal consistency of putative natural clusters and to examine the robustness of these clusters using bootstrapping methodologies. These extra steps will extend the investigation of the psychometric properties and robustness of the putative natural TAND clusters. We also acknowledge that the natural clusters were generated using only the TAND Checklist data. There may therefore be other natural clusters that could be identified using different kinds of fine-grain data. However, the purpose of the TAND Checklist was to provide a simple and easy-to-use tool for clinical practice. For this reason, we set out to examine the potential of the TAND Checklist data to generate natural TAND Clusters, given that such a strategy has a far greater potential for larger-scale implementation.

## Conclusion

In spite of the highly heterogeneous nature of TAND manifestations, the data-driven strategies used here in search of natural TAND clusters were able to replicate the findings from the feasibility study in a larger sample of children and adults with the pen-and-paper TAND Checklist data collected across seven countries. The study not only identified several similarities between the findings from the two data sets but also identified key aspects and next steps that will require larger-scale data, replication, and expansion. If these steps could replicate and extend the natural TAND clusters suggested in these preliminary studies, the natural TAND clusters may have the potential to help develop novel approaches to identification and treatment of TAND and may suggest novel data-driven strategies to subgroup individuals with TSC for clinical and research purposes.

## Data Availability

Novartis supports the publication of scientifically rigorous analysis that is relevant to patient care, regardless of a positive or negative outcome. Qualified external researchers can request access to anonymized patient-level data, respecting patient informed consent, contacting study sponsor authors. The protocol can be accessed through EnCePP portal http://www.encepp.eu/ (EU PAS Register Number EUPAS3247).

## Abbreviations

ADHD: Attention deficit/hyperactivity disorder
ASD: Autism spectrum disorder
TAND: TSC-associated neuropsychiatric disorders
TSC: Tuberous sclerosis complex
